# A New Genetic Algorithm Approach Applied to Atomic and Molecular Cluster Studies

**DOI:** 10.3389/fchem.2019.00707

**Published:** 2019-11-05

**Authors:** Frederico T. Silva, Mateus X. Silva, Jadson C. Belchior

**Affiliations:** ^1^Departamento de Química Fundamental-CCEN, Universidade Federal de Pernambuco, Cidade Universitária, Recife, Brazil; ^2^Programa de Pós-Graduação em Modelagem Matemática e Computacional, Centro Federal de Educação Tecnológica de Minas Gerais (CEFET-MG), Belo Horizonte, Brazil; ^3^Departamento de Química-ICEx, Universidade Federal de Minas Gerais, Belo Horizonte, Brazil

**Keywords:** cluster optimization, quantum genetic algorithm (QGA), evolutionary operator management, Lennard-Jones clusters, polynitrogen structure optimization

## Abstract

A new procedure is suggested to improve genetic algorithms for the prediction of structures of nanoparticles. The strategy focuses on managing the creation of new individuals by evaluating the efficiency of operators (*o*_1_, *o*_2_,…,*o*_13_) in generating well-adapted offspring. This is done by increasing the creation rate of operators with better performance and decreasing that rate for the ones which poorly fulfill the task of creating favorable new generation. Additionally, several strategies (thirteen at this level of approach) from different optimization techniques were implemented on the actual genetic algorithm. Trials were performed on the general case studies of 26 and 55-atom clusters with binding energy governed by a Lennard-Jones empirical potential with all individuals being created by each of the particular thirteen operators tested. A 18-atom carbon cluster and some polynitrogen systems were also studied within REBO potential and quantum approaches, respectively. Results show that our management strategy could avoid bad operators, keeping the overall method performance with great confidence. Moreover, amongst the operators taken from the literature and tested herein, the genetic algorithm was faster when the generation of new individuals was carried out by the twist operator, even when compared to commonly used operators such as Deaven and Ho cut-and-splice crossover. Operators typically designed for basin-hopping methodology also performed well on the proposed genetic algorithm scheme.

## 1. Introduction

Clusters are aggregates of atoms or molecules whose structures remain between those of discrete atoms and of the bulk material (Johnston, 2003). Moreover, their properties are composition and size dependent. Palladium, for instance, is non-magnetic in the solid state, but its counterpart clusters may have non-zero magnetic moment (Moseler et al., 2001). Among the wide range of interesting cluster applications one could mention magnetic resonance imaging (Lu et al., 2017), water oxidation (Zhao et al., 2017), magnetic storage (Bader, 2006), and catalysis (Pelegrini et al., 2016). In addition, clusters are promising in the development of nanomachines (Rieth and Schommers, 2002), Islas et al. and Merino et al., for example, showed the stability of boron wheels (Islas et al., 2007; Jiménez-Halla et al., 2010), while the latter researchers also studied the aromaticity of such particles and the rotational motion of these rings with respect to each other, comparing their behavior to a wankel motor (Jiménez-Halla et al., 2010). However, for most of computational chemistry techniques, atomic coordinates are needed for the calculation of clusters properties, and hence one must know the cluster structure. Finding the geometries of small clusters is a challenging task and requires a combination of theoretical and experimental techniques (Götz et al., 2012; Heiles et al., 2012).

It is generally assumed that clusters adopt the lowest energy structure (Lazauskas et al., 2017). Accordingly, finding such structure is a matter of finding the global minimum of an appropriate potential energy surface (PES). Modeling such PES within a quantum approach rapidly becomes computationally prohibitive, therefore empirical analytic expressions are usually employed to describe the interactions between the particles composing the clusters. Examples of these potentials are the Lennard-Jones (Jones and Ingham, 1925), Morse (Morse, 1929), and REBO (Brenner et al., 2002; Kosimov et al., 2010; Bonnin et al., 2019; Jiang et al., 2019; Lin et al., 2019) potentials, the latter being a more complex one which has gained prominence due to its applicability to describe graphene potential energy surfaces (Jiang et al., 2019; Lin et al., 2019).

Once the way to compute the energy of the system has been defined, one must minimize it. There are several techniques that enable global minima search, such as big bang methodology (BB) (Lazauskas et al., 2017), basin-hopping (BH) (Rondina and Da Silva, 2013) and evolutionary algorithms, such as genetic algorithms (GA) (Johnston, 2003). Especially, GAs have been successfully applied to predict chemical structures from clusters to protein folding (Johnston, 2003; Louis and McDonnel, 2004; Heiles et al., 2012; Silva et al., 2014b; Borguesan et al., 2015; Song et al., 2018). Even so, finding the global minimum associated with these chemical systems implies efficiently exploring the most reasonable portions of their PES, which still is a challenging task. Therefore, new algorithms are constantly being developed.

## 2. Related Work

It is already well discussed in the literature that, in order to guarantee efficiency in convergence and appropriate exploration of the PES associated with atomic and molecular clusters, evolutionary algorithms employed in global optimization problems must ensure population diversity (Hartke, 1999; Cheng et al., 2004; Grosso et al., 2007; Pereira and Marques, 2009; Marques et al., 2018). Therefore, estimating how similar are the structures composing the evolving population can provide valuable information to assist the evolutionary procedure. In the work of Hartke (1999), it is proposed that a minimum degree of exploration of the PES is ensured by making part of the population always composed by mutants. That means a set of structures that have been randomly modified will be present throughout the evolutionary procedure, regardless of whether they are better adapted or not. In the same work, a minimum energy difference between structures is established to maintain diversity, as well as a balance between optimization performance and exploration of the PES is proposed through the simultaneous use of a random operator such as Deaven and Ho (1995) cutting plane and a biased version of this operator in which the cluster is separated into its best and worst halves. Hartke (1999) also proposes a measure based on the two-dimensional projections of clusters structures that can distribute different types of geometries into niches. Thus, different ranges of values can be assigned to different types of geometries, allowing the evaluation of structure similarities and enabling one to avoid population stagnation.

Cheng et al. (2004) propose that structure similarity checking should always be based on topological information, and that measurements of the distance between energy minimum structures should be carried out by comparing numerical values associated with structure similarities. In their work, a connectivity table for cluster similarity checking is proposed, in which the connectivity information of a cluster is characterized according to the number of atoms having *i* nearest neighbors within the cluster. By using this connectivity table together with the evaluation of the fitness of each individual, they managed to balance diversity and convergence efficiency. Pereira and Marques (2009) state that one should consider structural information for estimating dissimilarities among cluster structures when searching for energy minima within an evolutionary algorithm approach, instead of taking into account fitness values. They have employed a combination of an evolutionary approach with a local search method that uses derivative information to search for the nearest local minimum without requiring any previous knowledge about the problem being solved. The authors show that maintaining diversity is the main issue to guarantee effectiveness, which was carried out by the application of three distinct distance measures to estimate the dissimilarity between structures.

As for recent advances in the development of genetic algorithms, Heiles et al. coupled Plane-Wave Self-Consistent Field (PWscf) package with Birmingham Cluster Genetic Algorithm (BCGA), allowing the study of Au-Ag nanoalloys through density functional theory (Heiles et al., 2012). Zayed et al. implemented what they called universal genetic algorithm, making use of Python's large collection of libraries and of the scaling capabilities of a pool genetic algorithm (Zayed et al., 2017). Vilhelmsen and Hammer proposed an inexpensive strategy to eliminate similar structures from the population (Vilhelmsen and Hammer, 2012). Lazauskas et al. proposed a pre-screening to eliminate structures with high probability of convergence failure during local minimization (Lazauskas et al., 2017).

In the past we proposed two new operators, namely annihilator and history operators (Guimarães et al., 2002), that demonstrated along the years (Lordeiro et al., 2003; Rodrigues et al., 2008; Silva et al., 2014a,b) to be quite efficient for determining global minima in atomic and molecular cluster studies where many local minima were present. Regarding the creation of new individuals, one can observe a broad variation among methodologies available in the literature. In general, each operator application rate is kept constant throughout the GA execution. For instance, Wang et al. used the values 0.5, 0.3, and 0.2 for mating, mutation and exchange rates, respectively, in their global minimization (Wang et al., 2018). Zhao et al. propose values between 10% and 30% for mutation rate (Zhao et al., 2016), while in an outline of the evolutionary principles of GAs, Heiles and Johnston describe a parameter that defines the probability of mutation, *p*_*mut*_ (Heiles and Johnston, 2013). Let *n*_*tot*_ be the total number of individuals to be created after energy minimization of an arbitrary generation; among them, *p*_*mut*_*n*_*tot*_ individuals are created by mutation operators, while (1−*p*_*mut*_)*n*_*tot*_ are created by crossover or recombination methods, on average (Heiles and Johnston, 2013). Finally, Rondina *et al*. used a dynamic strategy to manage operators in a basin-hopping technique (Rondina and Da Silva, 2013).

In this work, we propose a method with dynamic management of evolutionary operators for genetic algorithms that, in principle, could lead to a more efficient way to survey the PES of atomic and molecular clusters than our previous older GA version (Guimarães et al., 2002; Lordeiro et al., 2003). The paper is divided as follows: section 3 outlines a standard GA procedure, gives the details of our algorithm and describes all the operators employed as well as the management strategy proposed. The comparison between the different builds tested and the evaluation of their behavior according to the model system employed are presented and discussed in section 4. The main conclusions are gathered in section 5.

## 3. Methodology

### 3.1. Genetic Algorithm Procedure

A standard GA procedure is defined by three main steps. The first step is initialization, when an initial population of individuals is generated. The second step is selection, where all individuals are ranked according to their fitness and, in the present work, the 25% worst are eliminated. The third step is the creation of new individuals, where, in general, operators are applied to individuals that survived the selection step to generate new structures. We call operators all ways of generating a new member of the population. Desirable operators are the ones which efficiently span the potential energy surface of the system representatively. This can be done in different ways to which different concepts are associated and will be discussed further on. After creation step, the whole population is submitted to selection again and the cycle is repeated (Johnston, 2003).

One can find a wide variety of genetic algorithms in which the basic structure just described has been customized to improve performance or to meet some specific needs. In fact, the generation of the initial population may not always be completely random (Johnston, 2003; Chen et al., 2013); the measure of the quality of the individuals (fitness) might be given by different mathematical approaches (Burton and Vladimirova, 1998; Jin et al., 2002; Yan and Wang, 2010), and its upper and lower limits may be fixed or scaled in each generation according to the current population (Johnston, 2003). The selection of individuals to be eliminated or to generate offspring may depend on their fitness values in different ways, as well as various methods are available for choosing parents for mating (Saini, 2017). Furthermore, subpopulations can be evolved in parallel and exchange individuals along the procedure, simulating migration in natural populations (Chen et al., 2013). These few examples, and all their possible combinations, illustrate the versatility of genetic algorithms.

In this work, however, we concentrate mainly on the creation of new individuals within an approach focused on the study of atomic and nanoalloy clusters. Our approach changes the creation rate of each operator employed on the fly, favoring the better ones. In order to do so, we first performed a study over 13 evolutionary operators collected in the literature (Deaven and Ho, 1995; Michalewicz, 1996; Wales and Doye, 1997; Johnston, 2003; Takeuchi, 2007; Kim et al., 2008; Ye et al., 2011; Chen et al., 2013; Rondina and Da Silva, 2013) and evaluated the performance of all proposed builds within a 26 and 55-atom Lennard-Jones potential clusters (LJ_26_ and LJ_55_) approach and a simple evolutionary scheme.

**Figure 1:**
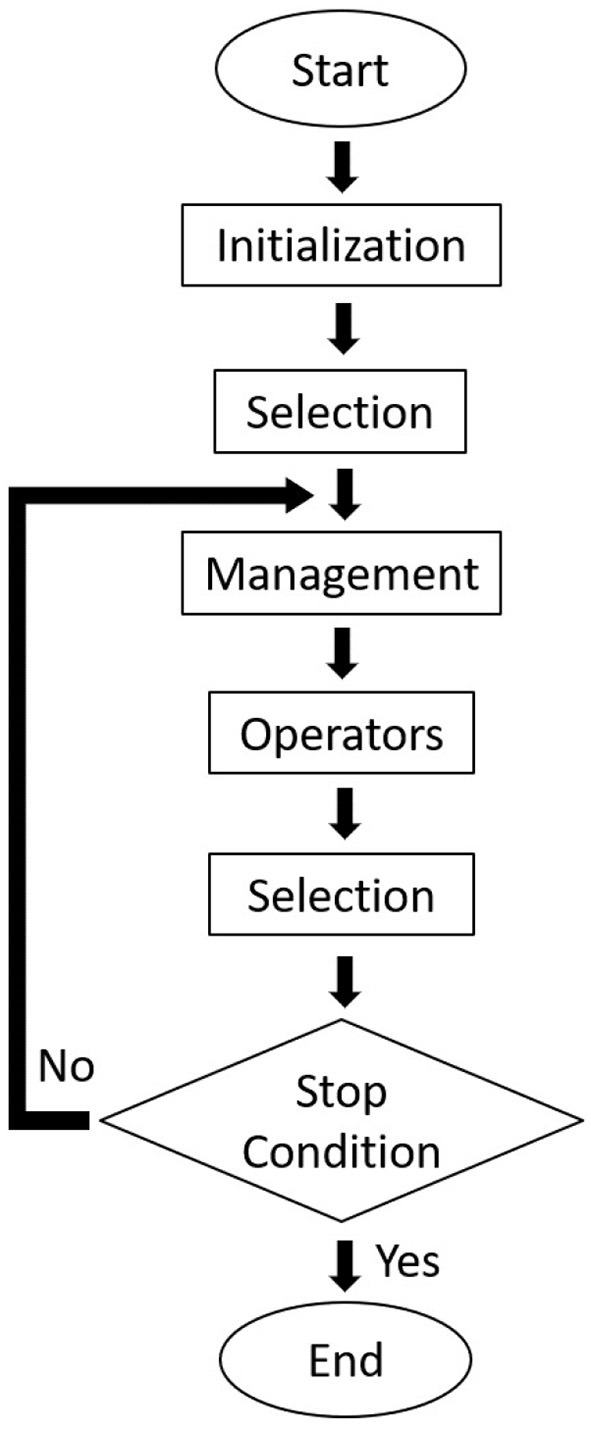
A flowchart of our genetic algorithm procedure.

We have employed a primary GA framework and focused on the outcomes of each operator, both individually tested and jointly implemented, when tackling simple systems such as LJ_26_ and LJ_55_. We have also briefly approached the harder LJ_38_ system, the C_18_ cluster employing the more complex REBO potential and applied our management strategy within a quantum approach to polynitrogen systems. The scheme of the genetic algorithm implemented in this work is presented in Figure 1, and each of its steps will be discussed in the following sessions. The program was written in C++ and the calculations were made on an Unix computer. For the polynitrogen cases, however, a more robust algorithm (Silva et al., 2018) was chosen (coupled to GAMESS-US, Schmidt et al., 1993). In the future we intend to both extend this approach to molecular nanoclusters and enhance the efficiency of our algorithm by improving each of its steps with typical strategies (Johnston, 2003) that help avoiding unnecessary computational effort and assist convergence.

### 3.2. Initialization

Following Cai et al. (2002), each individual of the initial population is created by randomly generating atoms inside a mathematical sphere of radius *R*, defined by Equation (1):

(1) $$\begin{array}{r} R=r_{e}\left[\frac{1}{2}+{\left(\frac{3N}{4\pi \sqrt{2}}\right)}^{\frac{1}{3}}\right] \end{array}$$

where *N* is the number of atoms and *r*_*e*_ is a parameter related to the equilibrium distance between atoms, here set to 1.0. Additionally, a restriction was added to prevent atoms from being generated very close to each other. The minimum distance allowed between two atoms at this step is 0.8 (dimensionless units adopted).

### 3.3. Selection and Stop Condition

In the present work, two analytic potentials were chosen to define the potential energy surfaces to be explored, namely the Lennard-Jones and REBO potentials. The adjustment of the parameters associated with the operators tested, as well as the evaluation of the employed builds were carried out using Lennard-Jones empirical potential (Equation 2) with reduced units (ϵ = σ = 1).

(2) $$\begin{array}{r} E=4\epsilon \underset{i<j}{\sum }\left[{\left(\frac{\sigma }{r_{ij}}\right)}^{12}-{\left(\frac{\sigma }{r_{ij}}\right)}^{6}\right] \end{array}$$

REBO potential was used further on to test the ability of the proposed builds to reach the global minimum in a more complex problem, the C_18_ cluster. This potential is described in detail in Brenner et al. (2002) and Kosimov et al. (2010), and it has been implemented with support from the Atomic Simulation Environment (ASE) library (Larsen et al., 2017). Among the options available in this simulation environment, we opted for the implementation of REBO present in Atomistica library. We have used dlib (King, 2009) library with limited-memory Broyden-Fletcher-Goldfarb-Shanno (L-BFGS) algorithm for local minimizations. In each generation, individuals were sorted by potential energy and the 25% worst were eliminated. From the remaining population, parent individuals are chosen for mating employing an uniform selection method, in which they are selected randomly uniformly from the current population. Individuals are also selected for mutation in the same manner. Although each operator has its own probability of acting over the population in each generation, it governs only the operator creation rate, but not the choice of an specifically ranked individual to act on.

**Figure 2:**
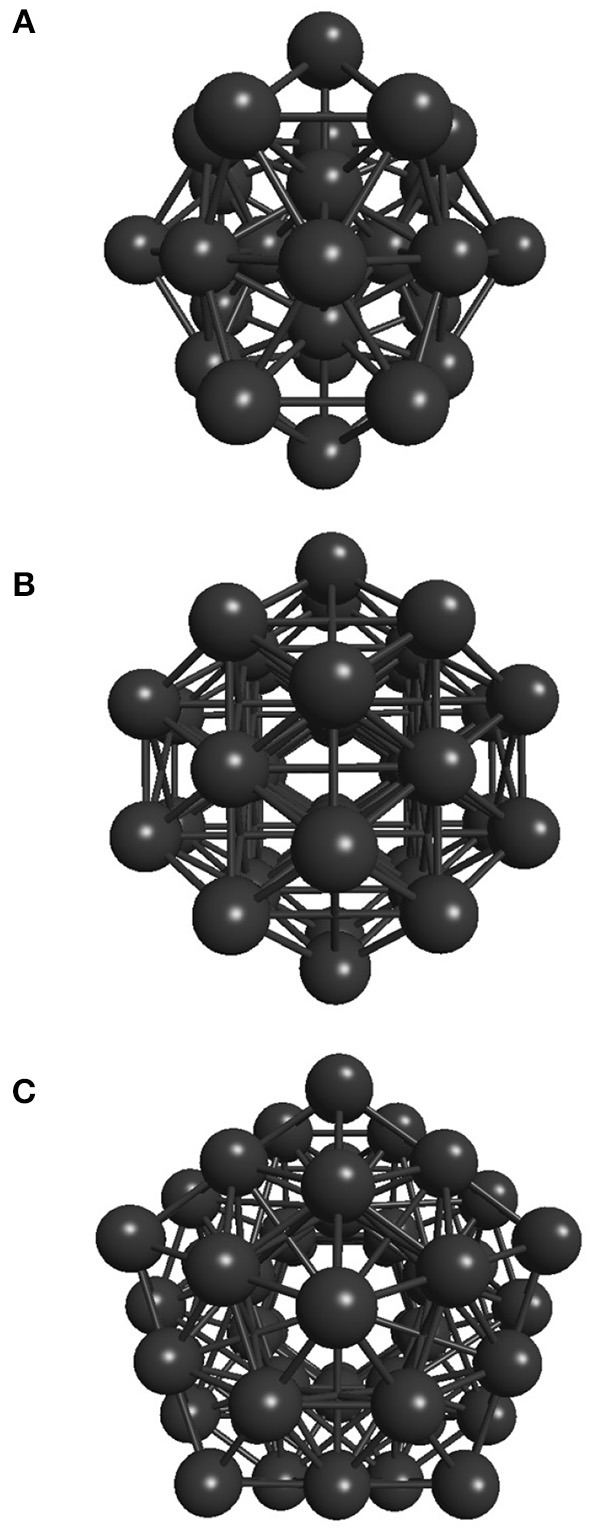
Well-known global energy minimum structures of the **(A)** 26-atom, **(B)** 38-atom, and **(C)** 55-atom Lennard-Jones clusters.

**Figure 3:**
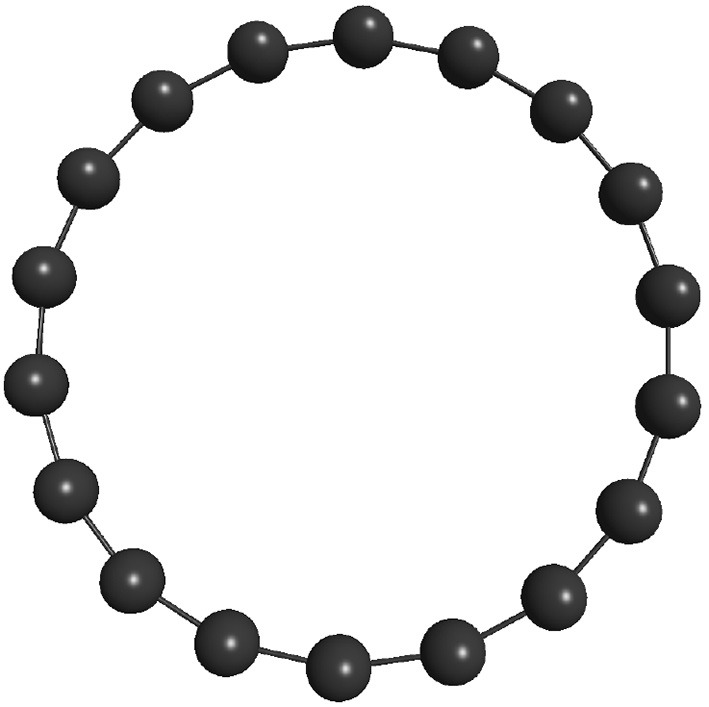
Most stable structure for C_18_ cluster: a planar single-ring (Kosimov et al., 2010).

Two stopping criteria were defined for the developed genetic algorithm. The first one is satisfied when the global minimum is achieved, which is already known for the Lennard-Jones 26, 38, and 55-atom cases studied here (−108.315616, −173.928427, and −279.248470, respectively), whose structures are shown in Figure 2. Also, the most stable structure for the 18-atom carbon cluster is known to be a planar monoring (shown in Figure 3), with binding energy equal to −108.3726 eV (Kosimov et al., 2010). The other criterion is fulfilled when 3,000 local minimizations are performed for C_18_ and the smaller Lennard-Jones cluster, and 5,000 local minimizations for the larger Lennard-Jones clusters. Usually, the global minimum is not known, and thus another termination criterion must be defined. For the polynitrogen cases tested, for example, the procedure is stopped either if it reaches 400 generations or if an individual remains as the one with the lowest energy for 20 consecutive generations (Silva et al., 2018). However, the former described stopping criteria are suitable for this work because performance was evaluated according to the number of local minimizations (*N*_*LM*_) needed to reach the global energy minimum. Therefore, after reaching this point (which is already known), additional calculations are not necessary. This performance assessment suggested here was also used by Chen et al. (2013) for the proposition of a new crossover operator, where a sphere is used to cut and splice the parent structures, rather than a plane.

### 3.4. Management

The method we propose to manage the application of operators within the evolutionary procedure is based on setting, on the fly, the creation rate of each operator employed according to their outcomes. When a new individual is generated, its energy is compared to the average energy of the entire population that survived the previous selection step. If the energy of the new individual is lower than this average energy, more individuals will be created with that operator in the next generation. If, on the other hand, the energy of the new individual is above that average, the related operator suffers a decrease in its creation rate. The function chosen to describe how the creation rate of each operator *o*_*j*_ changes along the evolutionary procedure (from the current to the next generation) is piecewise-defined:

(3) $$\upsilon_{ij}\left(\Delta E_{ij}\right)=\{\begin{array}{ll} \upsilon_{max}, & \Delta E_{ij}<-\Delta E_{max}\\ \left(\frac{\upsilon_{max}}{\Delta E_{max}}\right)\Delta E_{ij}, & -\Delta E_{max}<\Delta E_{ij}<\Delta E_{max}\\ -\upsilon_{max}, & \Delta E_{ij}>\Delta E_{max} \end{array}$$

where υ_*ij*_ is the i^th^ contribution to the variation of the creation rate of operator *j*. Δ*E*_*ij*_ is the energy difference between the new individual *i*, created by *o*_*j*_, and the average of the population that survived the previous selection step. The maximum allowed value for the variation of the creation rate of any operator employed in the algorithm was defined as υ_*max*_ and here set to 0.9. Δ*E*_*max*_, here set to 2.0, is the energy difference that triggers maximum variation. Thus, the new creation rate (*p*_*j*_) of each operator *o*_*j*_ is defined according to Equation (4):

(4) $$\begin{array}{r} p_{j}=p_{j}^{\prime }+\frac{1}{N}\overset{N}{\underset{i}{\sum }}\upsilon_{ij}\left(\Delta E_{ij}\right) \end{array}$$

where $p_{j}^{\prime }$ is the creation rate of operator *j* in the previous generation, and *N* is the number of individuals it has created in the current generation.

After all creation rates have been modified, their sum is normalized to one. Lastly, new individuals are created by the rule *p*_*j*_*n*_*tot*_, where *p*_*j*_ is the creation rate of operator *j* and *n*_*tot*_ is total number of individuals that must be created.

### 3.5. Operators

Traditionally, the creation of new individuals is done in two different manners: through crossover or mutation (Johnston, 2003). Crossover combines two individuals from the population to produce new ones, simulating the combination of genetic information from the parents to generate offspring. Mutation modifies the coordinates of a single individual from the population to generate a new one, avoiding population stagnation. It simulates the introduction of new genetic material to the population. In this work, three types of operators are used: crossover, which produces a new individual combining other two; mutation, which produces a new individual from a single one; and immigration, which creates a new individual from scratch, simulating migration in natural environment.

The following operators are of crossover type. They take two individuals (*k* and *l*) from the remaining population, chosen randomly from the group of the previous selection step survivors, to generate a new one (*m*).

- Arithmetical crossover (ARCR) (Michalewicz, 1996): let *x*^(*k*)^ be the cartesian coordinate vector of individual *k*, *x*^(*l*)^ the cartesian coordinate vector of individual *l* and *x*^(*m*)^ the cartesian coordinate vector of the new cluster *m*. ARCR acts to generate a new individual with the following rule: *x*^(*m*)^ = 0.5(*x*^(*k*)^ + *x*^(*l*)^).
- Plane-cut-splice crossover (PCCR) (Deaven and Ho, 1995): a plane is randomly defined separating the atoms of cluster *k* into two groups. Another random plane is defined for cluster *l*, also separating its atoms into two groups. The groups generated from cluster *k* must have the same number of atoms of those generated from cluster *l*. Then, equivalent groups are exchanged between clusters *k* and *l* to generate the new cluster *m* with the correct number of atoms.
- Sphere-cut-splice crossover (SCCR) (Chen et al., 2013): analogous to PCCR, but using a sphere instead of a plane. A mathematical sphere is defined to separate cluster *k* into two groups of atoms, one that lies in the inner part of the sphere and other that lies in its outer region. The same sphere is generated for cluster *l*. If the inner part of both progenitor clusters contains the same number of atoms, they are interchanged to generate a new individual *m*.
- Two points crossover (TWCR) (Johnston, 2003): the coordinates of the atoms composing each of the selected individuals for mating must be arranged in a one-dimensional array. Then, two random integers are generated: *s*_1_ = [1, (3*N*−1)] and *s*_2_ = [(*s*_1_+1), 3*N*]. The notation [*a, b*] means that a random number between *a* and *b*, in a uniform distribution, must be generated. The coordinates of cluster *k* that lie between *s*_1_ and *s*_2_ are replaced by those of cluster *l* that lie on the same range.
- Uniform crossover (UNCR) (Johnston, 2003): when generating the new individual *x*^(*m*)^, each new coordinate $\left(x_{i}^{\left(m\right)}\right)$ has a specific probability of coming from each of its parents. In the present approach the new individual has 70% chance of coming from cluster *k* $\left(x_{i}^{\left(k\right)}\right)$ and 30% chance of coming from cluster *l* $\left(x_{i}^{\left(l\right)}\right)$.

The following operators are of mutation type. They take one individual, *k*, also chosen randomly from selection step survivors, to generate a new one, *m*.

- Angular operator (AO) (Wales and Doye, 1997): this operator acts on 1–5% of the total number of atoms in the cluster, chosen randomly. Each selected atom is displaced randomly over the surface of a sphere of radius *R*_*i*_ (equal to the distance of the atom to the geometric center of the cluster) centered in the geometric center of the particle.
- Cartesian displacement operator (CDO) (Rondina and Da Silva, 2013): this operator acts on 1 to *N* atoms, chosen randomly. *N* is the total number of atoms in the cluster. Each selected atom is modified by the following equation:
  (5) $$\begin{array}{l} r_{i}^{\left(m\right)}=r_{i}^{\left(k\right)}+Sr_{min}([-1,+1\;]\hat{\;\text{i}}\;+\;[-1,\;+\;1\;]\;\hat{\text{j}}\;\\ \;+\;[-1,\;+\;1\;]\;\hat{\text{k}}\;) \end{array}$$
  where $r_{i}^{\left(m\right)}$ are the new coordinates of the cluster's *i*^*th*^ atom, $r_{i}^{\left(k\right)}$ are the former coordinates of that same atom, *S* is an arbitrary parameter, here set to 0.2, $\hat{\text{i}}$, $\hat{\text{j}}$ and $\hat{\text{k}}$ are the cartesian unit vectors, and *r*_*min*_ is the distance to the nearest atom to which the operator will act. Again, the notation [−1, +1] means that a random number with uniform distribution must be generated between −1 and +1.
- Dynamic mutation (DYM) (Johnston, 2003): this operator acts on all atoms of the selected individual according to the following equation:
  (6) $$\begin{array}{r} r_{i}^{\left(m\right)}=\left[\left(1-\delta \right),\left(1+\delta \right)\right]r_{i}^{\left(k\right)} \end{array}$$
  where $r_{i}^{\left(m\right)}$ are the new coordinates of the cluster's *i*^*th*^ atom, $r_{i}^{\left(k\right)}$ are the former coordinates of that same atom and δ is an arbitrary parameter, here set to 0.10.
- Geometric center displacement operator (GCDO) (Kim et al., 2008; Rondina and Da Silva, 2013): this operator acts on 1 to *N* atoms, chosen randomly. *N* is the total number of atoms in the cluster. Each selected atom is modified by the following equation:
  (7) $$\begin{array}{l} r_{i}^{\left(m\right)}=r_{i}^{\left(k\right)}\;+\;[(\alpha_{max}-\alpha_{min}){\left(\frac{R_{i}}{R_{max}}\right)}^{w}\\ \;+\alpha_{min}]\;r_{min}\;{\hat{e}}_{i}\;(\theta_{i},\varphi_{i}) \end{array}$$
  where $r_{i}^{\left(m\right)}$ are the new coordinates of the cluster's *i*^*th*^ atom, $r_{i}^{\left(k\right)}$ are the former coordinates of that same atom, *r*_*min*_ is the distance between the *i*^*th*^ atom and its nearest neighbor, *R*_*i*_ is the distance between the *i*^*th*^ atom and the geometric center of the particle, *R*_*max*_ is the distance between the center of the particle and its furthest atom, α_*max*_, α_*min*_ and *w* are arbitrary parameters, here set to 0.2, 0.7, and 2.0, respectively, and ê_*i*_(θ_*i*_, φ_*i*_) is a unit vector generated randomly in a spherical distribution.
- Interior operator (IO) (Takeuchi, 2007; Ye et al., 2011): this operator moves a single atom toward the particle's nucleus. Let *R*_*i*_ be the distance between the *i*^*th*^ atom and the geometric center of the particle. Atom *i* is moved to a random position on the surface of a sphere of radius [0.01, 0.10]*R*_*i*_, centered on the geometric center of the particle.
- Surface angular operator (SAO) (Ye et al., 2011): this operator moves a single atom toward the surface of the cluster. Let *R*_*max*_ be the distance between the geometric center of the particle and its furthest atom. Selected atom, *i*, is moved to a random position on the surface of a sphere with radius *R*_*max*_, centered on the geometric center of the particle.
- Twist operator (TO) (Johnston, 2003; Rondina and Da Silva, 2013): a random plane is defined to separate the selected cluster into two portions, not necessarily with the same sizes. Then, one of these portions is rotated randomly around the axis formed by the normal to that plane. In this work, the angle of rotation, θ, was generated randomly between 0.10π and 0.50π.

The following operators are of immigration type. They create a new individual, *m*, from scratch.

- Immigration (IMM and IMM0) (Cai et al., 2002): this operator generates a new individual in the same manner the initial population is created. Namely, atoms are generated randomly inside a sphere of radius defined by Equation (1). Two types of immigration are defined: IMM and IMM0. IMM has a restriction that prevents atoms from being created closer than 0.8Å to each other. IMM0 does not have any restriction.

### 3.6. Test Methodology

In order to implement this new methodology for a GA based on the management of various mathematical operators, several builds were designed using the operators just described, individually and combined. Several tests were performed as well. All tests followed the same protocol in which the genetic algorithm was executed 50 times with different random number seeds. As described in section 3.3, the chosen systems were the general case studies of 26 and 55-atom clusters with binding energy governed by a Lennard-Jones empirical potential (LJ_26_ and LJ_55_) with ϵ = σ = 1 (reduced units). Two stopping conditions were used: finding the global minimum or reaching 3,000 local minimizations for LJ_26_ or 5,000 local minimizations for LJ_55_. The cluster population was kept constant in 40 individuals, 10 of them being eliminated at each generation and replaced by the available creation operators. The initial population was randomly generated using the method described in section 3.2.

Three different builds were proposed and used to test the management methodology described in section 3.4, namely AUTO5, AUTO7, and AUTO13. The numbers indicate how many creation operators were employed in each build. AUTO5 is composed by the following operators: TO, IO, PCCR, SAO, and IMM. AUTO7 is composed by: TO, IO, PCCR, SAO, IMM, AO, and GCDO. Finally, AUTO13 build is composed by all the operators tested herein: TO, IO, PCCR, SAO, IMM, AO, GCDO, TWCR, CDO, SCCR, UNCR, ARCR, and DYM. We have also run the same build of our previous work, here named PREV, which is composed of 70% SCCR, 20% DYM, and 10% IMM, kept fixed throughout the GA execution (Silva et al., 2015). Operator acronyms were defined in section 3.5.

## 4. Results and Discussion

Results yielded by all builds tested are presented in Table 1 for the LJ_26_ case. $\hat{N_{LM}}$ is the average number of local energy minimizations needed to achieve convergence to the global minimum, $\sigma_{x}^{-}$ is the standard error, defined as the standard deviation divided by the square root of the total number of samples, and *N*_*fails*_ is the percentage of seeds employed that did not achieve convergence for a specific build. For those cases that failed to converge *N*_*LM*_ was defined as the maximum allowed number of local minimizations plus one (3001). However, unconverged runs were not taken into account to obtain $\hat{N_{LM}}$. The results are presented primarily in ascending order of *N*_*fails*_; secondarily, in ascending order of $\hat{N_{LM}}$.

**Table 1:** Results of tests performed on our GA builds for LJ_26_.

**Build**	$\hat{{\text{N}}_{\text{LM}}}$^*^	$\sigma_{x}^{-}$	***N_fails_(%)***
TO	186	17	0
IO	205	16	0
PCCR	246	32	0
AUTO5	246	39	0
AUTO7	264	27	0
SAO	264	23	0
IMM	297	39	0
AO	272	33	2
AUTO13	434	61	2
GCDO	357	66	4
PREV^**^	720	120	6
UNCR	1,096	135	12
TWCR	634	62	16
CDO	739	113	16
SCCR	371	71	28
IMM0	1,242	197	52
ARCR	390	71	80
DYM	3,001	0	100

*$\hat{N_{LM}}$ indicates the average number of local minimizations needed to reach the global minimum. $\sigma_{x}^{-}$ indicates the standard error and N_fails_ indicates the relative number of times the global minimum was not reached*.

* *The unconverged runs were removed from the calculation of these averages. This removal may compromise the analysis when N_fails_ is nonzero*.

** *Previous work Silva et al. (2015)*.

We call attention to builds TO, IO, PCCR, AUTO5, AUTO7, SAO, and IMM, which managed to find the global minimum on every run. On the other hand, DYM was the only one that failed to properly converge on all test runs with different values assigned to the δ parameter. Acting on all atoms of the selected individual at once seems to be an ineffective mutation for our purpose.

Twist operator (TO) was the one with lowest $\hat{N_{LM}}$, however, it overlaps with interior operator (IO) if we take their standard errors into account. Within the same analysis, standard errors show that IO performed similarly to PCCR and AUTO5, which in turn were essentially equivalent to AUTO7 and SAO. Since the global minimum of LJ_26_ is approximately of spherical shape, it favored interior (IO) and surface angular (SAO) operators, explaining their good performances. In order to compare our top ranked build (TO) with the widely used plane-cut-splice (PCCR), which did not overlap considering their standard errors, we have used one-tailed *p*-value approach (Chaubey, 1993) and calculated that the twist operator build was better than plane-cut-splice crossover build with a 90% confidence level. To ensure that this comparison would be valid, we had previously tested for the normality of the data generated by these builds using the Ryan-Joiner test (Yap and Sim, 2011), and the normality hypothesis was accepted within a significance level of 0.01 with less than five percent of discrepant data removed. The PCCR proposed by Deaven and Ho (1995), however, still had a good performance, since its build managed to find the global minimum on every run and presented one of the lowest $\hat{N_{LM}}$ values. This operator is employed in most of modern genetic algorithms (Johnston, 2003; Heiles and Johnston, 2013) and had its robustness already reevaluated, showing good results (Froltsov and Reuter, 2009).

The geometric center displacement operator (GCDO) presented better performance than the cartesian displacement operator (CDO). The parameters associated with each of these methods were refined before final test in both cases. The better GCDO performance could be explained by the two additional parameters available for tuning compared to CDO. The uniform crossover (UNCR), two points crossover (TWCR) and arithmetical crossover (ARCR) were not originally developed for cluster studies, and, among them, TWCR was the one that presented the best performance. They make up the worst performing group within the LJ_26_ approach along with CDO, SCCR, IMM0, and DYM.

Still for the LJ_26_ case, the sphere-cut-splice crossover (SCCR) performed poorly, which was expected since Chen et al. indeed reported that this operator is more suitable for larger clusters (Chen et al., 2013). In our previous work (Silva et al., 2015), the employed build (PREV) was mainly composed by SCCR, but also counted with the immigration operator and a different evolutionary scheme. Within the present GA approach, our PREV build presented worse performance ($\hat{N_{LM}}=720$) than SCCR-only build ($\hat{N_{LM}}=371$) when it comes to the average number of local minimizations needed to reach global minimum. Nevertheless, the PREV build presented better reliability than SCCR-only on finding the correct cluster structure, since the failure rate of the latter (28%) was almost five times greater than that of the former (6%). If we took the unconverged runs into consideration to calculate ${\hat{N_{LM}}}^{*}$, PREV would go from $\hat{N_{LM}}=720$ to ${\hat{N_{LM}}}^{*}$ = 857, while SCCR would go from $\hat{N_{LM}}=371$ to ${\hat{N_{LM}}}^{*}$ = 1107. The improvement of our PREV build over the SCCR-only could be explained by the joint presence of the IMM operator in the PREV build, which had better performance here and was responsible for the creation of 10% of *n*_*tot*_ in each generation in our previous work. The immigration operator (IMM) itself was the fifth most efficient in the present study. However, it is important to note that the restriction that prevents atoms from being created too close to each other was decisive in its performance. Without such restriction, $\hat{N_{LM}}$ goes from 297 (IMM) to 1242 (IMM0). Besides, IMM0 failed to converge on 52% of the trials within the LJ_26_ approach.

In our PREV build we were focused on the development of a GA to be coupled with electronic structure methods. Therefore, we needed to generate reasonable structures from the very beginning, while keeping population diversity within an unbiased analysis. That is because bad structures may easily lead to unconverged energy calculations or local minimizations in a quantum approach, unlike the empirical potential case, in which the energy may always be obtained analytically. On the other hand, avoiding completely stochastic contributions to the evolutionary procedure could prevent us from finding new energy minima, typically hard to guess if one has no previous information about the system. Seeking for good cost-effectiveness relation was essential to survey the *ab initio* potential energy surface associated with atomic clusters without calling upon empirical potentials. However, that was a difficult task to fulfill employing specific operators with fixed creation rates.

**Figure 4:**
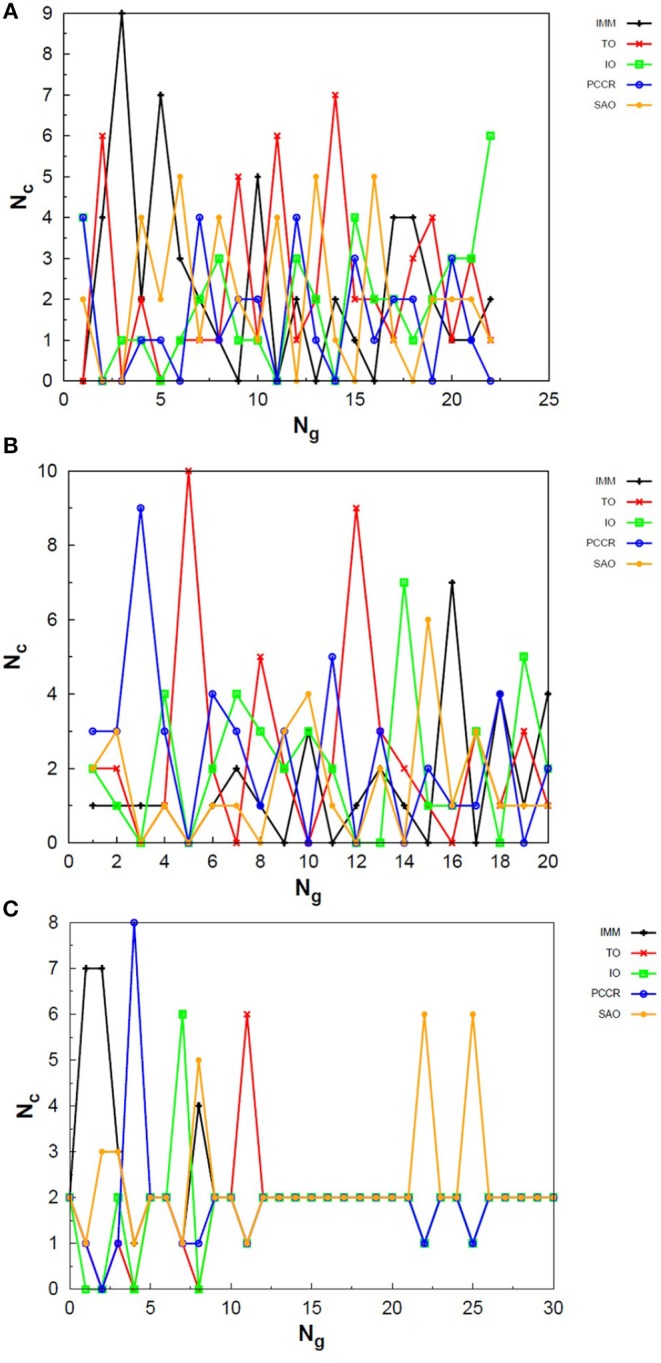
Evolution of the creation of new individuals for AUTO5 build throughout three different runs of the LJ_26_ system, corresponding to the **(A)** third, **(B)** thirty-ninth and **(C)** ninth random number seed employed. *N*_*c*_ is the number of individuals created with that operator and *N*_*g*_ is the generation index. In general, the graphs show large variation in creation rates in the first generations and smaller variations at the end of the simulations.

**Figure 5:**
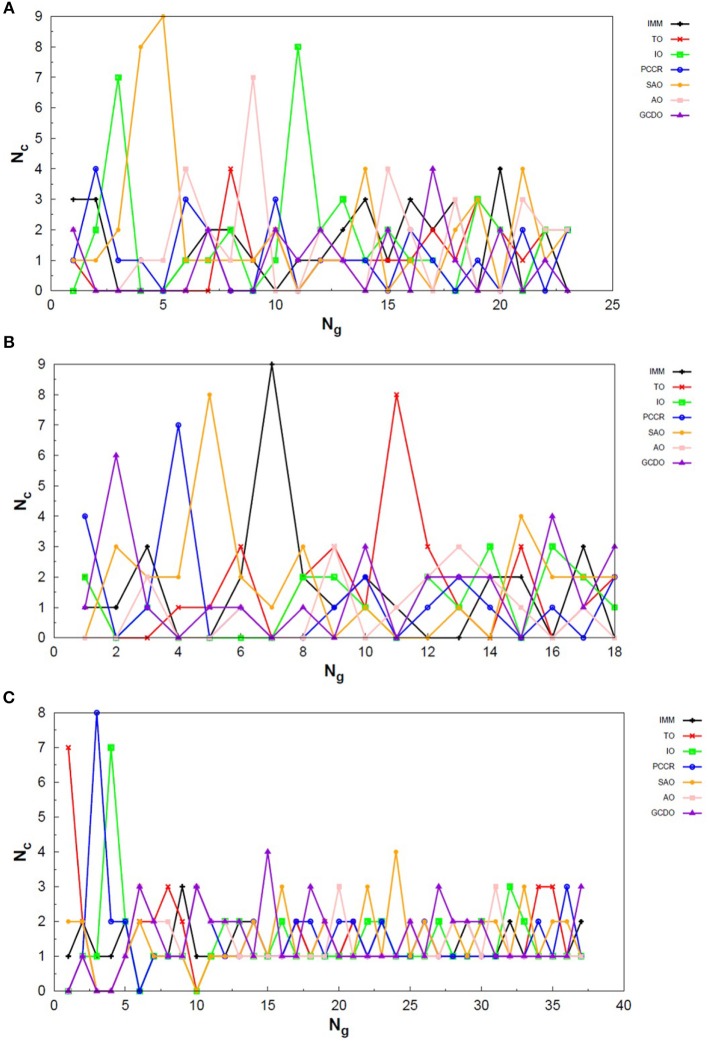
Evolution of the creation of new individuals for AUTO7 build throughout three different runs of the LJ_26_ system, corresponding to the **(A)** thirty-seventh, **(B)** forty-fourth and **(C)** ninth random number seed employed. *N*_*c*_ is the number of individuals created with that operator and *N*_*g*_ is the generation index. In general, the graphs show large variation in creation rates in the first generations and smaller variations at the end of the simulations.

**Figure 6:**
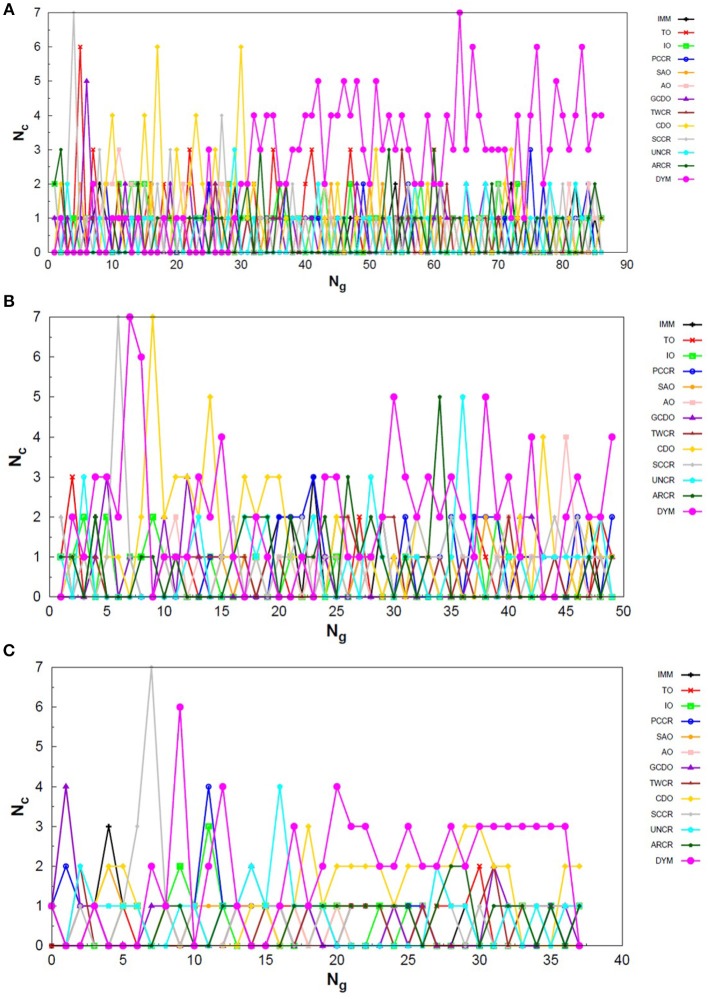
Evolution of the creation of new individuals for AUTO13 build throughout three different runs of the LJ_26_ system, corresponding to the **(A)** seventeenth, **(B)** thirty-ninth and **(C)** ninth random number seed employed. *N*_*c*_ is the number of individuals created with that operator and *N*_*g*_ is the generation index. Except for the peculiar behavior of the DYM operator, the graphs show generally larger variation in creation rates in the first generations and smaller variations at the end of the simulations. The number of generations to reach the global minimum was, on average, greater than that required for the other builds.

Exploring different possibilities of combining these evolutionary operators together may provide more flexibility to the algorithm and hence allow more thorough sampling of the PES in a single run. In the first instance, we are mostly interested in evaluating solely the contribution of the operators to the general performance of genetic algorithms. From this perspective, we can evaluate the behavior of our AUTO5, AUTO7, and AUTO13 builds within the highly unbiased GA scheme adopted here, in which the simplest rules were used to generate the population, to rank it and to select individuals for mating and mutation, as well as for predation. Through the graphs presented in Figures 4–6, we can assess the variations in the creation rate of each operator within our management strategy along different GA runs (chosen randomly) concerning the LJ_26_ system. Figure 4 refers to AUTO5, Figure 5 to AUTO7 and Figure 6 to AUTO13.

In general, these creation rates undergo great variations during the first generations and converge to smaller oscillations within a narrower range as the process advances. This can be better noticed when the number of generations needed to reach the global minimum is greater, such as in Figure 5C. In the graphs of Figure 4 one can also notice the importance of TO to the AUTO5 build, which indeed was the responsible for the largest average creation rate in several runs of that build concerning the LJ_26_ system. Still about AUTO5, it is interesting to note that some operators seem to be more important at different stages of the evolutionary procedure, while others seem to be more systematic. In Figures 4A,C it can be seen that the creation rate of IMM, for example, is larger at the beginning and decreases as the system evolves, while essentially the opposite behavior can be observed for IO in Figures 4A,B. IMM creates individuals totally randomly, and thus it could be expected to yield better results in a stage where the population is not sufficiently evolved. IO and TO, in turn, were the ones that presented the best performances when evaluated individually within the GA scheme employed here to approach the LJ_26_ system, which is consistent with their behaviors within AUTO5 build.

Among the builds proposed to test our management methodology, AUTO7 seems to be the most balanced one. For the simple LJ_26_ case, for example, its performance has been essentially equivalent to that of AUTO5, as one can see from $\hat{N_{LM}}$ in Table 1 and from the number of generations needed to reach convergence, shown in Figure 5. Besides, it has presented, on average, more homogeneous creation rates among its operators throughout the generations when compared to AUTO5 and AUTO13. AUTO7 collects not only the operators that presented the best results when evaluated individually, but also those that failed to converge for some of the random number seeds tested, despite presenting comparable good performance according to $\hat{N_{LM}}$ (and considering the standard errors). Along with keeping overall performance, this combination allowed a desirable diversity of operator outcomes. Furthermore, this specific combination of operators could enhance the performance of individual ones, such as SAO, which presented the largest average creation rate in two out of the three runs shown in Figure 5.

From the comparison between Figures 4–6 one can also notice that AUTO13 generally required a considerably larger number of generations to reach global minimum than AUTO7 and AUTO5, which was already expected due to the results shown in Table 1. Excluding the DYM operator, the graphs in Figure 6 also show generally larger variations in creation rates in the first generations and more steady behavior in later generations. However, by analyzing the graphs in Figure 6, we can conclude that the presence of operators that performed badly when evaluated individually indeed contributed to the worse performance of AUTO13. Operators such as CDO, UNCR, and DYM managed to create individuals good enough to raise their creation rates, but not sufficiently good to reach the global minimum. These inadequate operators undermined the action of the most suitable ones to perform the original task of finding the lowest energy structure effectively. This means that AUTO13 frequently got stucked in local minima and probably would not be the best choice to tackle a system for which not much information is already available.

A study completely analogous to that presented so far was carried out for the 55-atom case (LJ_55_) and the results yielded by the builds tested are presented in Table 2. Only the builds that successfully converged in more than 50% of the trial runs are presented. For those cases that failed to converge *N*_*LM*_ was defined as the maximum allowed number of local minimizations plus one (5001). Again, unconverged runs were not taken into account to obtain $\hat{N_{LM}}$. The results are presented primarily in ascending order of *N*_*fails*_; secondarily, in ascending order of $\hat{N_{LM}}$.

**Table 2:** Results of tests performed on our GA builds for LJ_55_.

**Build**	$\hat{{\text{N}}_{\text{LM}}}$^*^	$\sigma_{x}^{-}$	***N_fails_(%)***
IO	559	32	0
TO	559	53	0
GCDO	571	41	0
AUTO7	653	37	0
AUTO5	660	46	0
PCCR	775	42	0
AO	830	60	0
SAO	1,380	109	0
AUTO13	864	74	4
TWCR	1,419	190	28
CDO	1,724	223	44

*$\hat{N_{LM}}$ indicates the average number of local minimizations needed to reach the global minimum. $\sigma_{x}^{-}$ indicates the standard error and N_fails_ indicates the relative number of times the global minimum was not reached*.

* *The unconverged runs were removed from the calculation of these averages. This removal may compromise the analysis when N_fails_ is nonzero*.

The results obtained for LJ_55_ are essentially consistent with those obtained for the LJ_26_ case. IO and TO were again the ones with best performances and, along with GCDO, AUTO7, AUTO5, PCCR, AO, and SAO, make up the builds that managed to find the global minimum on every run. Among the ones proposed to test our management strategy, AUTO7 and AUTO5 performed equivalently again and, once more, outperformed AUTO13. Just as done in the 26-atom case, we have also compared our top ranked builds (IO and TO) with the widely used plane-cut-splice (PCCR) for the 55-atom case using one-tailed *p*-value approach (Chaubey, 1993). This time, our automated builds (AUTO5 and AUTO7) did not overlap with PCCR when taking their standard errors into account, thus we have also compared AUTO7 (which was essentially equivalent to AUTO5) with PCCR using one-tailed *p*-value approach. Again, we have previously tested for the normality of the data generated by these builds using the Ryan-Joiner test (Yap and Sim, 2011), and the normality hypothesis was accepted within a significance level of 0.1 without data discard. IO and TO were better than PCCR with a 99% confidence level, while AUTO7 was better than PCCR with a 95% confidence level.

For this larger system, TWCR, CDO, UNCR, ARCR, and DYM performed even worse than they did for the LJ_26_ case, as expected due to the increase in difficulty to find the global minimum as the number of degrees of freedom of the system increases. This time, however, IMM also performed badly and could not converge a significant amount of runs. On the other hand, AO and GCDO did not fail in any run as they did in the previous case. Again, SCCR performed badly, although it was expected to improve when approaching larger systems (Chen et al., 2013).

Although we have separated operators into classes (crossover, mutation, and immigration) in section 3.5, no distinction was made among them when it comes to the number of individuals created by each type in each generation. This was always set on the fly according to our management strategy described in section 3.4 (or kept fixed for the builds with single operators). As a result, mainly mutation type operators presented good performances within our GA approach, both individually and within the builds composed by various operators. Furthermore, operators that act fully in a random way performed generally better than more complex ones which involve, for example, parameterized mathematical expressions or simply parameters to be defined. The latter may be more suitable for less unbiased GA schemes than the one employed here. Excepting PCCR, crossover operators were greatly outperformed, possibly because they need more elaborate methods to select parents for mating to properly yield results. ARCR and SCCR, for instance, did not present high values for $\hat{N_{LM}}$, but they did present high *N*_*fails*_. This indicates that these operators might be more sensible to the fitness of the selected parents. Finally, regarding the IMM operator, it is reasonable to expect that it would perform worse for larger systems, since the probability of randomly generating good structures would undoubtedly decrease with the number of atoms.

The management strategy proposed in this work proved to be efficient. The performance of AUTO5, for example, approached the average taken over the performance of its individual operators (TO, IO, PCCR, SAO, and IMM) for the LJ_26_ case. This was measured by taking the average value of $\hat{N_{LM}}$ over the five cited operators, which equals 240, while $\hat{N_{LM}}$ associated with AUTO5 was 246. For AUTO7 ($\hat{N_{LM}}=264$) we have a similar scenario: the average of $\hat{N_{LM}}$ over its individual operators equals 261. On the other hand, AUTO13 ($\hat{N_{LM}}=434$) considerably outperformed the average of its operators ($\hat{N_{LM}}=620$) and, furthermore, presented only 2% of convergence failure, despite being composed by various operators that showed high failure rate.

For the LJ_55_ case, some operators that presented high values of *N*_*fails*_ when employed individually were still added to AUTO5, AUTO7, and AUTO13 builds. In order to compare the performances of these builds with those of their individual operators, however, only the ones shown in Table 2 were taken into consideration. Thus, for AUTO5 ($\hat{N_{LM}}=660$) it was measured by taking the average value of $\hat{N_{LM}}$ over IO, TO, PCCR, and SAO, that equals 818; for AUTO7 ($\hat{N_{LM}}=653$), the average value of $\hat{N_{LM}}$ was taken over IO, TO, GCDO, PCCR, AO, and SAO, which equals 779; for AUTO13 ($\hat{N_{LM}}=864$), the average value of $\hat{N_{LM}}$ was taken over IO, TO, GCDO, PCCR, AO, SAO, TWCR, and CDO, which equals 977. For the larger LJ_55_ cluster, all AUTO builds outperformed the average of their operators. These numbers would favor even further the AUTO builds if the operators omitted from Table 2 had been taken into account. This time, despite having individually ineffective operators in their compositions, all AUTO builds managed to enhance the overall performance.

We have also attempted to perform the same study for LJ_38_. However, it has a double funnel energy landscape (Chen et al., 2013) and hence is a more complicated system to be approached by our simple GA scheme. Therefore, none of our builds managed to converge to the global energy minimum more than 50% of the 50 trial runs. Builds such as ARCR, UNCR and DYM, for example, failed 100% of the trials, while AUTO5 was the best build and managed to find the LJ_38_ global minimum 48% of the times. This is because four out of the five operators that compose AUTO5 build were the ones that presented the highest individual convergence rates. In fact, they were even more effective than AUTO7 and AUTO13. It is interesting to notice, however, that the remaining AUTO5 operator, SAO, failed 94% of the times for the LJ_38_ case. It shows that, despite contaminated by an operator that performed badly individually, AUTO5 managed to outperform every other build when approaching the LJ_38_ system. Once more we have evidence that our management strategy may enhance the overall performance of the method through a synergic action of suitable operators.

**Figure 7:**
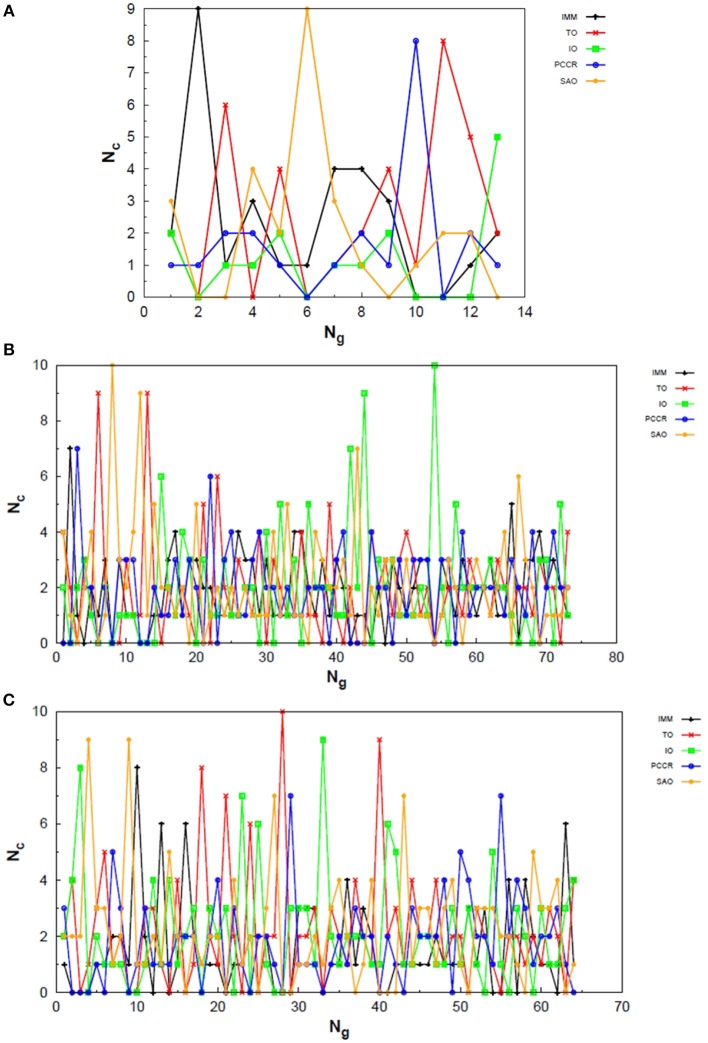
Evolution of the creation of new individuals for AUTO5 build throughout three different runs of the LJ_55_ system, corresponding to the **(A)** seventeenth, **(B)** thirty-ninth and **(C)** forty-fourth random number seed employed. *N*_*c*_ is the number of individuals created with that operator and *N*_*g*_ is the generation index. TO stands out again among the operators of AUTO5 build, followed by SAO and IO.

**Figure 8:**
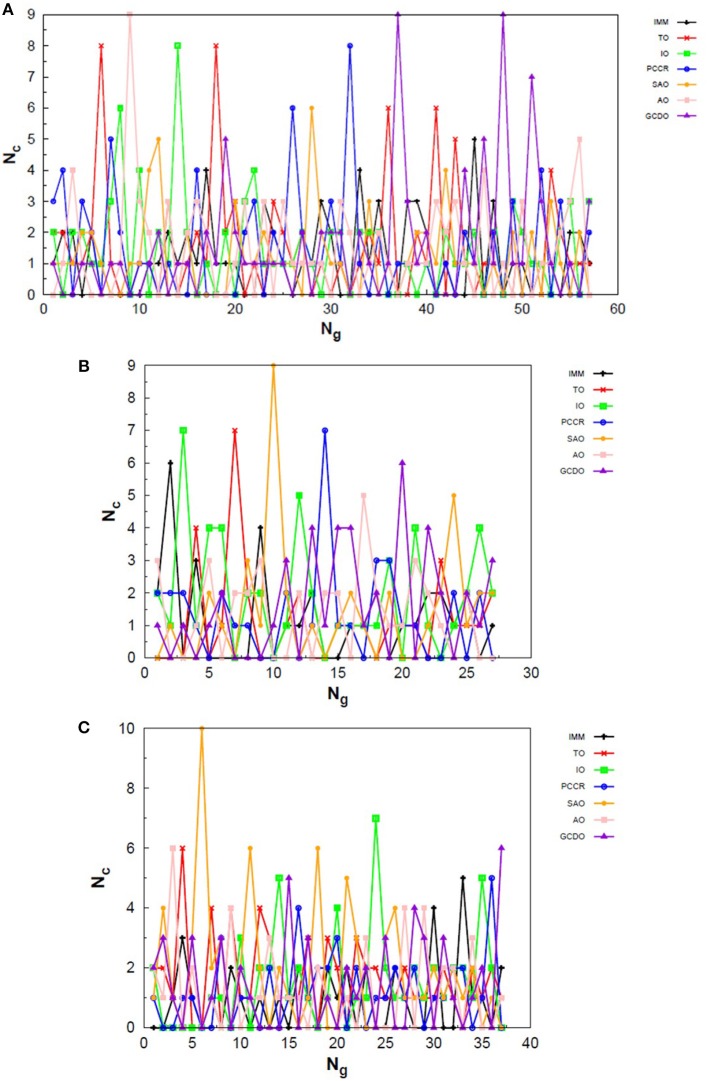
Evolution of the creation of new individuals for AUTO7 build throughout three different runs of the LJ_55_ system, corresponding to the **(A)** third, **(B)** seventeenth and **(C)** thirty-ninth random number seed employed. *N*_*c*_ is the number of individuals created with that operator and *N*_*g*_ is the generation index. AUTO7 was also the most balanced build for the LJ_55_ case, presenting wider diversity of operator outcomes.

**Figure 9:**
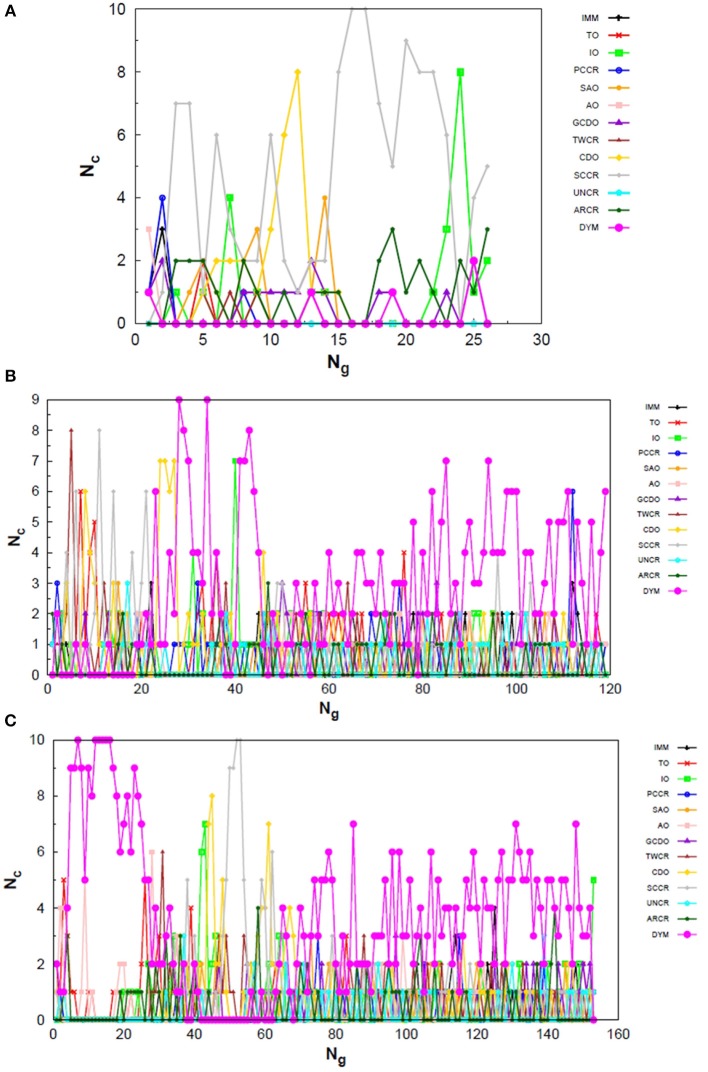
Evolution of the creation of new individuals for AUTO13 build throughout three different runs (randomly chosen) of the LJ_55_ system. *N*_*c*_ is the number of individuals created with that operator and *N*_*g*_ is the generation index. In **(A)** the DYM operator does not act significantly and convergence is reached quickly. In **(B,C)**, again, the DYM operator disturbs the evolutionary procedure causing the number of generations needed to reach the global minimum to be greater than that required for the other builds, on average.

Analogously to the LJ_26_ case, we can also assess the variation in the creation rate of the operators within each AUTO build along different GA runs (chosen randomly) regarding the LJ_55_ system. These results are shown in Figure 7 for AUTO5, Figure 8 for AUTO7 and Figure 9 for AUTO13.

For the 55-atom cluster one can still see greater variations in the creation rate of AUTO5 operators up to half of the generations of the runs shown in Figures 7B,C. In the same figure (mainly in panels a and c) it can be noticed the same trend observed for the IMM operator when approaching LJ_26_ with AUTO5 build: it has higher creation rates at the beginning and it gets lower as the system evolves. Again, TO was the responsible for the largest average creation rate for this build, which can be inferred from the graphs of Figure 7. This is also consistent with the results presented in Table 2, where TO appears as the one with best performance when approaching LJ_55_, along with IO. The interior operator, however, has the third largest average value for the creation rate in this case, being overcome, surprisingly, by SAO. This exemplifies that the combination of operators may enhance their individual performance. From the graphs of Figure 7 one can also note that SAO influenced mainly the initial stages of the presented runs.

AUTO7 was the most balanced build for LJ_55_, as well as it was for LJ_26_. It presented more homogeneous distribution of peaks throughout the generations in the graphs of Figure 8 when compared to the other AUTO builds. None of its operators has been systematically the one with the greatest average creation rate within the evaluated runs. AUTO7 has also required less generations than AUTO5 and AUTO13, on average, to reach convergence, as it can be seen by comparing the graphs in Figures 7–9. From the same comparison, we can see that AUTO13 was again the build that generally required more generations to find the global minimum.

Through the analysis of Figures 9B,C, it becomes clear that DYM operator disturbed the evolutionary procedure and prevented these runs from converging earlier. In fact, Figure 9 shows three distinct scenarios: in (a) SCCR dominates the process from the beginning and leaves no room for DYM. Accordingly, the GA converges in only 26 generations. In (b) DYM also starts with low creation rate, but it increases rapidly within a few generations and basically dominates the process from the 24^*th*^ generation on. The GA converges after 119 generations. In (c) DYM already starts with high creation rate and, although it oscillates and goes through a minimum for approximately 20 generations, it increases again and dominate the process until convergence is reached after 153 generations. As well as it happened to the 26-atom case, the algorithm has spent several generations trapped in local minima while the unsuitable DYM operator prevents other operators from acting and reestablishing the needed population diversity. By all means, it is interesting to notice that AUTO13 managed to find the correct LJ_55_ structure mainly under the influence of operators that failed almost 100% of the times they were tested individually.

In Table 3 one can find the average number of local minimizations needed to reach the lowest energy structure of C_18_, as well as the failure rate (*N*_*fails*_) of each build employed here to tackle the C_18_ system within the REBO potential approach. This failure rate indicates the relative number of times the global minimum was not reached by our algorithm. For the C_18_ case, this minimum corresponds to the carbon atoms arranged in a planar single ring (Kosimov et al., 2010). This cyclocarbon molecule was indeed synthetized by Kaiser et al. using atom manipulation by eliminating carbon monoxide from a cyclocarbon oxide molecule, and characterized by high-resolution atomic force microscopy (Kaiser et al., 2019).

**Table 3:** Average number of local minimizations needed to reach the global minimum ($\hat{N_{LM}}$) for the C_18_ cluster together with the failure rate (*N*_*fails*_) for each employed build in reaching that minimum.

**Build**	$\hat{{\text{N}}_{\text{LM}}}$^*^	***N_fails_(%)***
PCCR	1,815	26
AUTO7	1,667	34
AUTO5	2,073	50
AO	1,964	60
AUTO13	1,420	60
GCDO	1,175	62
IO	1,757	66
TO	1,735	86
CDO	1,065	88
TWCR	2,120	92
SCCR	908	94
PREV^**^	1,505	96
IMM	3,001	100
SAO	3,001	100
ARCR	3,001	100
UNCR	3,001	100
DYM	3,001	100

*For all cases, 50 different random number seeds were executed employing the same parameters of the previous studied systems*.

* *The unconverged runs were removed from the calculation of these averages. This removal may compromise the analysis when N_fails_ is nonzero*.

** *Previous work (Silva et al., 2015)*.

We observed that operators leading geometries toward spherical shape were disadvantaged. TO, for example, which had performed well in the previous cases, performed poorly in the present one. That is because torsions would take atoms off the plane, which is not consistent with the energy minimum for the present system, which is planar. IMM, for instance, which generates atoms randomly inside a sphere, could not reach the global minimum in any run, as one could expect. SAO operator is also biased to generate spherical structures, and could not find the planar energy minimum in any run. Analogous argument can be used for the poor performance of SCCR and PREV, for example. On the other hand, the best results for the C_18_ case were obtained by the PCCR build, probably due to the use of planes to slice each parent cluster in crossover step.

The builds proposed in the present work (AUTO5, AUTO7, and AUTO13) presented essentially the same behavior observed in the previous cases, in which the stability of results is maintained, benefiting the best operators and avoiding the worst ones for each specific case. Our management strategy provides the useful advantage of versatility to the optimization algorithm. Despite the best performances for the cases tested here have been obtained by builds composed by individual operators, the AUTO builds can make the algorithm more adaptable to a wider range of problems. Therefore, we believe that our management strategy would be useful to improve the exploration of PES associated with more complex systems when applied together with a more robust GA framework, which consists the next step of our research.

Based on the analysis carried out so far, we have chosen AUTO7 to apply our strategy within a quantum approach. In order to do so, we have incorporated this build to a more robust GA scheme, namely QGA, which was also coupled to GAMESS-US (Schmidt et al., 1993) and adapted to approach dissociative systems. We have already used QGA to approach polynitrogen systems and to predict good candidates for high energy density materials (HEDM) (Silva et al., 2018), and now we intend to run QGA with AUTO7 (referred to as QGA-7 from now on) to reproduce some energy minima found in our previous work and to evaluate the behavior of the operators along the generations. These polynitrogens are atomic nitrogen clusters, which means that they form structures with nitrogen atoms connected to each other mainly by single or double bonds. Therefore, these clusters consist of local energy minima on the PES, while the global minimum consists of the dissociated system into N_2_ molecules.

**Figure 10:**
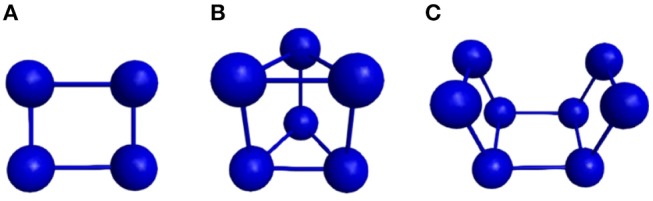
Local energy minima correctly found by QGA-7 for **(A)** N_4_, **(B)** N_6_, and **(C)** N_8_.

**Figure 11:**
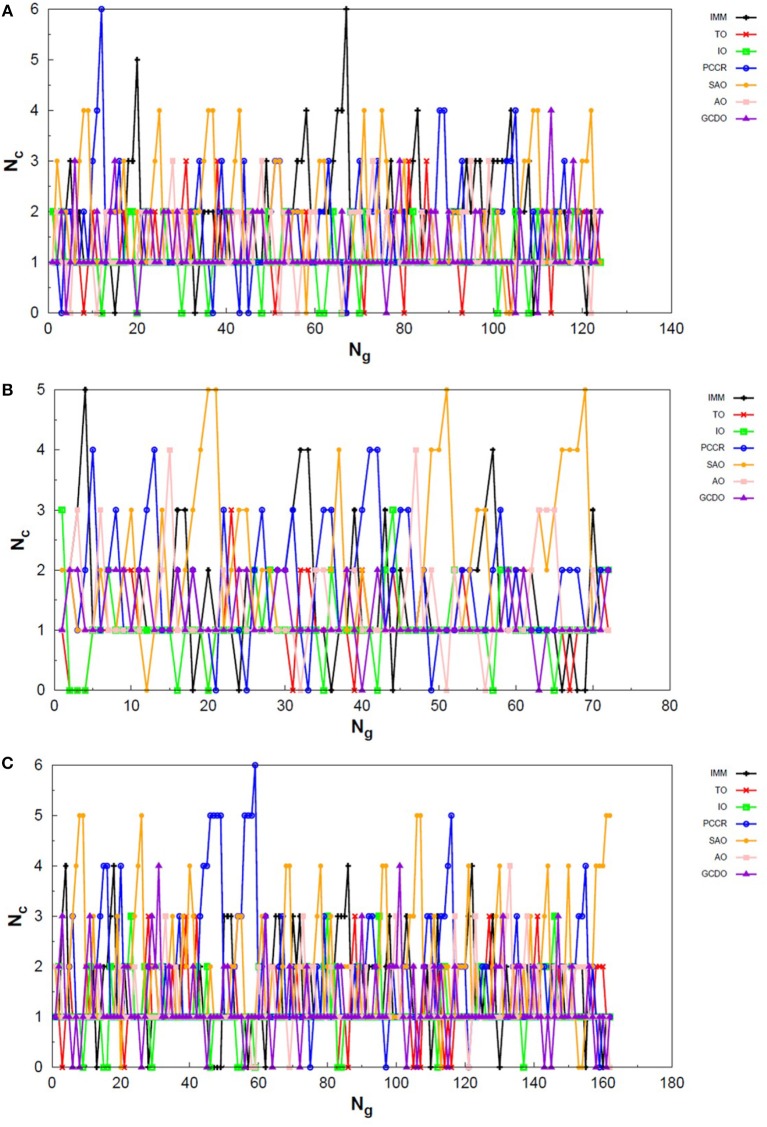
Evolution of the creation of new individuals for QGA-7 throughout three different runs of the N4 system, corresponding to the following random number seeds employed: **(A)** 17, **(B)** 29 and **(C)** 6217. *N*_*c*_ is the number of individuals created with that operator and *N*_*g*_ is the generation index.

**Figure 12:**
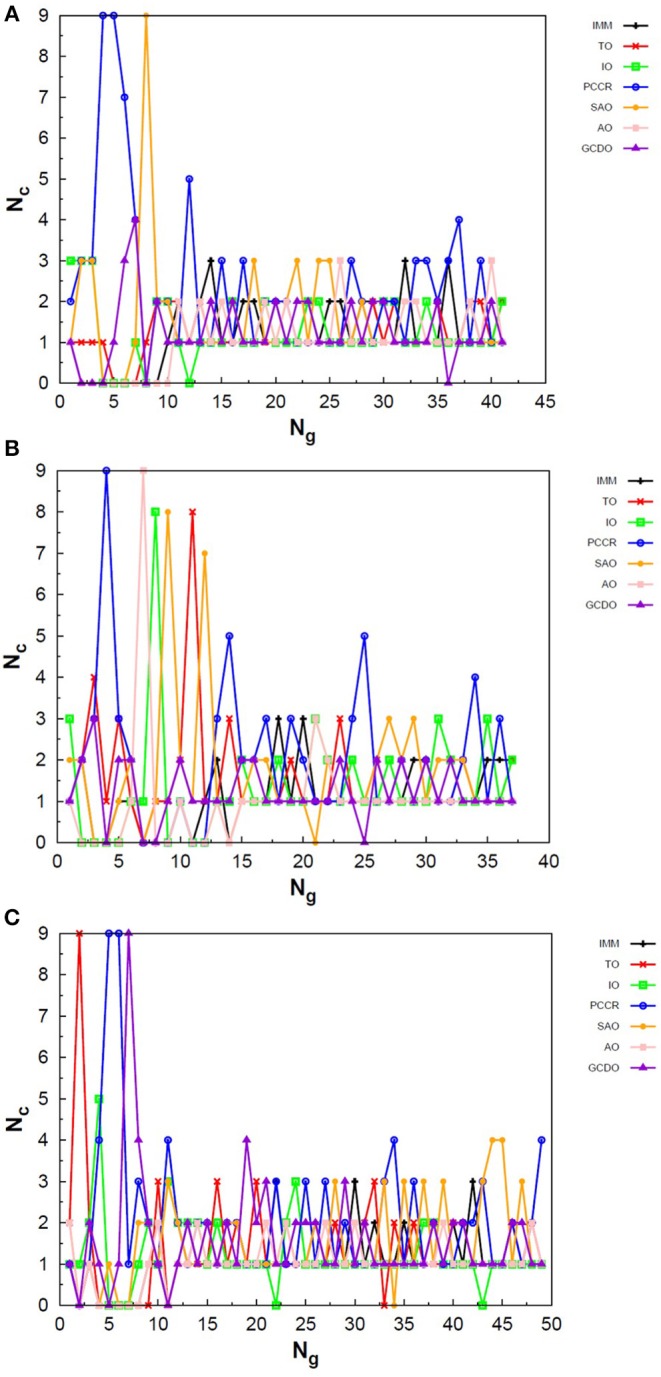
Evolution of the creation of new individuals for QGA-7 throughout three different runs of the N_6_ system, corresponding to the following random number seeds employed: **(A)** 9, **(B)** 29 and **(C)** 6217. *N*_*c*_ is the number of individuals created with that operator and *N*_*g*_ is the generation index.

**Figure 13:**
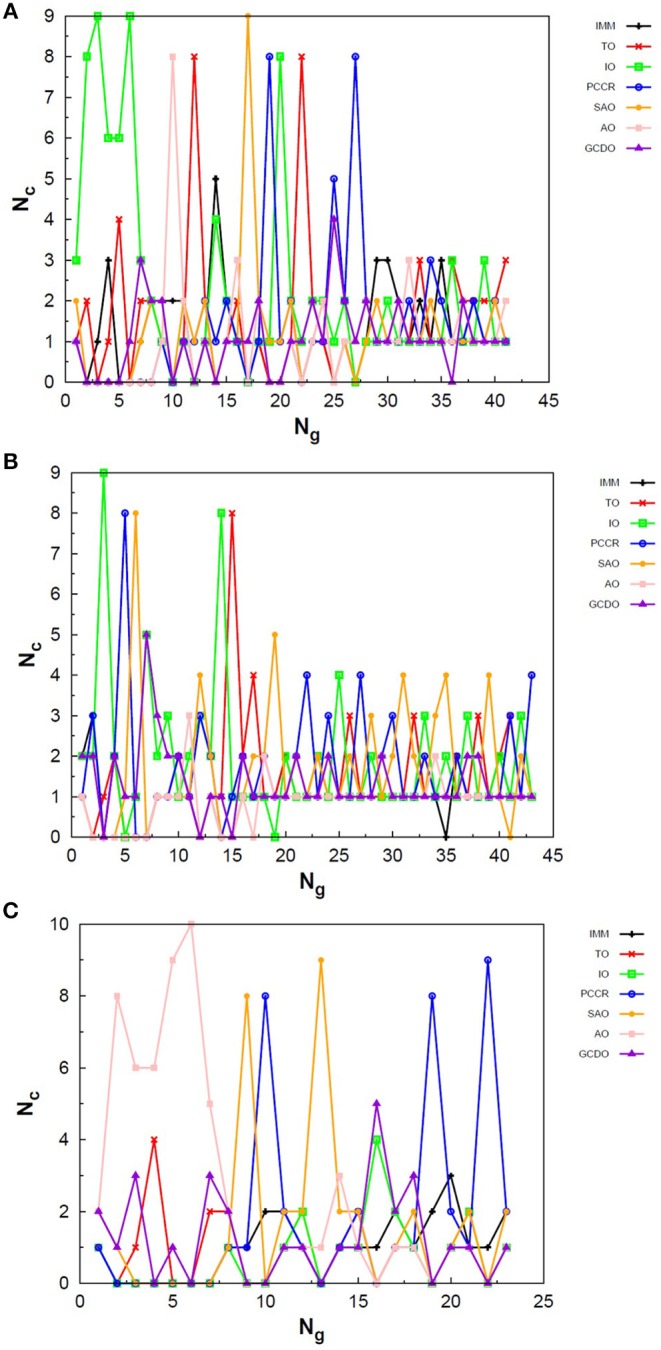
Evolution of the creation of new individuals for QGA-7 throughout three different runs of the N_8_ system, corresponding to the following random number seeds employed: **(A)** 29, **(B)** 62 and **(C)** 666. *N*_*c*_ is the number of individuals created with that operator and *N*_*g*_ is the generation index.

In Figure 10 one can find the structures corresponding to local energy minima on the PES associated with N_4_, N_6_ and N_8_ that we managed to reproduce with QGA-7 within a DFT approach employing B3LYP exchange and correlation functional and 6-31G basis set. Besides that, the variation in the creation rate of the operators within QGA-7 along different GA runs (chosen randomly) regarding the N_4_ system is shown in Figure 11. The same analysis concerning the N_6_ and N_8_ systems are presented in Figures 12, 13, respectively.

The graphs in Figure 11 show almost periodic oscillations in the creation rate of each operator involved in QGA-7. These oscilations have essentially constant amplitude for each operator over the entire evolutionary procedure, and are also quite homogeneous among the different ones. This, along with the large number of generations needed to reach convergence for such a small system, indicates stagnation. This can be explained by the fact that the D_2h_ structure (Figure 10A) for tetranitrogen is not that far from the much more stable 2N_2_ system within a random structure generator scheme perspective. Since QGA-7 was prepared so as not to allow these N_2_ fragments to get too far apart from each other, several quasi-degenerate structures may be generated before the optimum distance between these two moieties is reached. Nevertheless, QGA-7 still converged within the criterium established (an individual remained as the one with the lowest energy for 20 consecutive generations), and the operators that stood out, on average, were SAO, PCCR and IMM.

Differently from the behavior presented by the N_4_ system, the creation rate of the operators for the N_6_ and N_8_ cases resembled that observed for the LJ_26_ system, with greater variations along the first generations which stabilize to become smaller oscillations until convergence is reached. In fact, QGA-7 found the correct structures much more efficiently for these cases than for tetranitrogen. If we do not take into consideration the noisy initial part of the evolutionary procedure, it can be seen that, in general, the operators that stood out were PCCR and SAO. It is interesting to notice that SAO had an important role both in the Lennard-Jones and in the quantum approach of atomic clusters, while PCCR stood out mainly within the quantum approach and TO and IO stood out mainly within the classical approach.

Although we performed only a few simple tests with QGA-7, our management strategy applied to a more complex GA scheme and within a quantum approach was consistent with our primary tests on Lennard-Jones clusters. The results obtained so far may guide us toward the next steps to improve our algorithms, incorporate more efficient builds and enhance its performance to approach more complex systems.

Some well-identified problematic cases [such as LJ_38_, LJ_75−77_, LJ_98_, LJ_102−104_ and some short-ranged Morse clusters (Hartke, 1999; Cheng et al., 2004; Pereira and Marques, 2009)] must still be properly addressed in order to ensure that our strategy is indeed effective in exploring potential energy surfaces in a more extensive way. To do so, it is interesting that our algorithm become independent of extra information about the problem and less system-specific, that is more versatile, while maintaining population diversity (Lee et al., 2003; Grosso et al., 2007). We are currently implementing a new step in which all structures involved in each generation will be compared to each other so that we can evaluate structure similarities and avoid population stagnation. Within this future approach, even enantiomers may be told apart, and population diversity will be greatly enhanced. Different rules for the variation of the application rate of operators will also be tested, and not only the energy of the offspring may be compared to the average energy of the previous population, but also the capability of the applied operator to generate diverse structures. Furthermore, employing a less deterministic selection step, together with a combination of crossover and mutation operators to generate descendants may be also essential to help our algorithm tackle these harder optimization scenarios.

Nevertheless, the management strategy proposed in this work has already proved to be quite promising. Despite some single operator builds have performed better than the management methodologies tested (AUTO) for the LJ_26_ and LJ_55_ cases, this may not hold for larger and more complex systems, as well as for *ab initio* or DFT-based genetic algorithms. We propose that greater versatility might be essential to efficiently sample the PES and to avoid stagnating into a population with serious (SCF or structure optimization) convergence problems, mainly in the first generations.

## 5. Conclusions

We have developed a method that manages the creation rate of evolutionary operators within a genetic algorithm procedure on the fly. Its performance was evaluated on 26 and 55-atom Lennard-Jones clusters (LJ_26_ and LJ_55_) and the obtained results show that our strategy proved to be quite efficient. Moreover, we have assessed thirteen operators available in the literature and, within our simple and highly ubiased GA approach, twist operator was faster than commonly used Deaven and Ho's plane-cut-splice crossover. Also, interior and surface operators, formerly designed for basin-hopping methodology (Takeuchi, 2007; Ye et al., 2011), performed well in our genetic algorithm scheme, although they may have been favored due to the essentially spherical shape of the global energy minima approached.

Three different builds were proposed to test our management strategy, namely AUTO5, AUTO7, and AUTO13, where the numbers indicate how many creation operators were employed in each build. For the LJ_26_ case, the performances of AUTO5 and AUTO7 approached the average taken over the performances of their individual operators. This was measured by taking the average value of $\hat{N_{LM}}$ (average number of local energy minimizations) over the cited individual operators. On the other hand, AUTO13 considerably outperformed the average of its operators and, furthermore, presented only 2% of convergence failure, despite being composed by various operators that showed high failure rate.

For the LJ_55_ case, some operators that presented high failure rates when employed individually were still added to AUTO5, AUTO7, and AUTO13 builds. However, in order to compare the performances of these builds with those of their individual operators, only the latter ones that successfully converged in more than 50% of the trial runs were taken into consideration. Following this protocol, all AUTO builds outperformed the average of their individual operators. The numbers would favor even further the AUTO builds if all the individual operators tested had been taken into account, regardless of their failure rates. This time, despite having individually ineffective operators in their compositions, all AUTO builds managed to enhance the overall performance.

When tackling the C_18_ system, which presents a planar ring structure as the lowest energy minimum, we could observe that operators that relied mainly on spherical-based creation or transformations of individuals performed poorly, as one could expect. Our management strategy benefited the most appropriate operators and avoided the worst ones, making the algorithm more adaptable and versatile.

These results indicate that our management strategy could benefit from the advantages of the employed operators without loosing overall performance. It may actually enhance the overall performance and help to better explore the parameter space through the diverse combinations of appropriate evolutionary operators and efficient genetic algorithm schemes.

When approaching systems where the global minimum is not known, it is generally hard to tell which operator is the most suitable or efficient to promote GA convergence. Thus, employing several techniques combined and properly managing their application throughout the evolutionary procedure could be the best approach. Among the proposed builds, AUTO7, which combines diversity with speed, was the one chosen to be incorporated in a more robust GA scheme to test our strategy within a quantum approach to polynitrogen systems. This application of our management strategy was consistent with our simpler approach involving Lennard-Jones clusters. We have also managed to find correct polynitrogen structures and to evaluate the behavior of the creation rate of the operators involved in the proposed build within the quantum approach.

With the results yielded by this study we may be able to improve our builds by combining more appropriate operators, as well as our genetic algorithm itself, by implementing more efficient steps that could lead to faster convergence. This would be useful for further cluster studies, which may include *ab initio* and DFT potential energy surface survey.

## Data Availability Statement

All datasets generated for this study are included in the article/supplementary material.

## Author Contributions

FS: writing the code for the presented operator management strategy, including the employed genetic algorithm itself, as well as reviewing the literature for choosing the operators implemented in the algorithm and writing the paper. MS: running calculations to test the algorithm and the management strategy within the chosen Lennard-Jones systems, compiling, interpreting and evaluating the results, proposing needed modifications both to the employed strategy and to the final algorithm, taking the study to a quantum approach to polynitrogen systems and writing the paper. JB: guiding the studies, revising the paper and managing laboratory resources and research funding.

### Conflict of Interest

The authors declare that the research was conducted in the absence of any commercial or financial relationships that could be construed as a potential conflict of interest.
